# Supplementary figures and images for: A single base pair substitution in zebrafish distinguishes between innate and acute startle behavior regulation
Source: PLoS One. 2024 Mar 18;19(3):e0300529. doi: 10.1371/journal.pone.0300529 (PMC10947677; doi:10.1371/journal.pone.0300529)

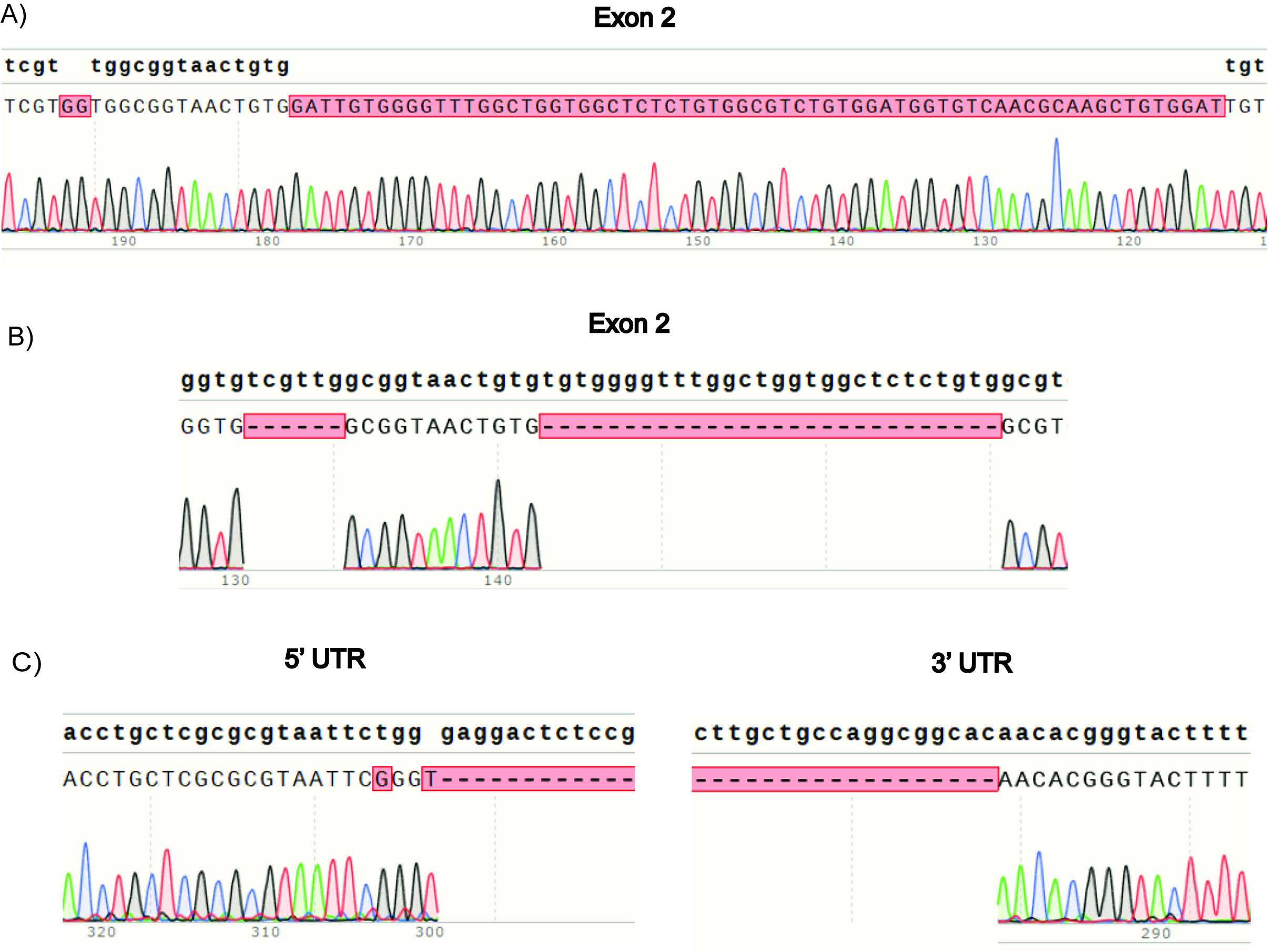

Supplement: S1 Fig — Chromatographs for A) p431 (syt7a67bpinsert), B) p432 (syt7a34bpdel), and C) p433 (syt7awldel) aligned to syt7a wildtype sequence. The wildtype syt7a sequence is indicated with lowercase bold letters. The mutated sequence is indicated with uppercase letters. The mutations of p431 and p432 are within exon 2 of the syt7a gene. The mutations for p433 are within the 5’UTR and 3’UTR of the syt7a and excise the entire coding syt7a sequence between these locations. (TIF) [file pone.0300529.s001.tif]

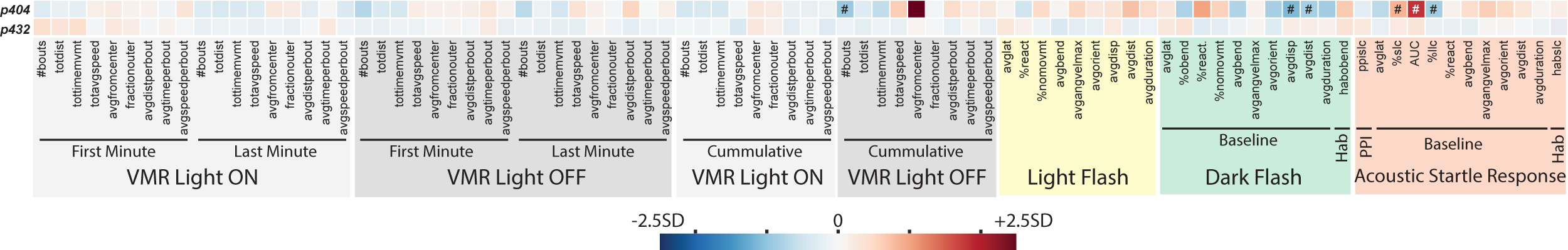

Supplement: S2 Fig — a) Heat map summary of the results from our broad behavioral assay for escapist (p404) line and syt7a34bpdel (p432) line. Different behavioral assays are designated by block: VMR Light ON (light gray box), VMR Light OFF (dark gray block), Light Flash (yellow block), Dark Flash (green block), and Acoustic Startle Response (red block). Each box within a block represents a different parameter tested during each assay. Responses from homozygous mutants were normalized to responses from their respective siblings (WT and Hets combined). The colors of the heatmap represent the Z-score, or the difference between the normalized average response from homozygous mutants compared to the average responses of their respective siblings in standard deviations, with red signifying mutants had a larger response than siblings and blue signifying mutants had a smaller response than siblings. A student t-test was used with a Bonferroni correction to correct for the multiple comparisons performed in the broad behavioral assay. Parameters that had a significant p-value after the Bonferroni correction are indicated with the pound sign (#) within the box. (TIF) [file pone.0300529.s002.tif]
